# Patient-derived models: Advanced tools for precision medicine in neuroblastoma

**DOI:** 10.3389/fonc.2022.1085270

**Published:** 2023-01-19

**Authors:** Kristina Aaltonen, Katarzyna Radke, Aleksandra Adamska, Alexandra Seger, Adriana Mañas, Daniel Bexell

**Affiliations:** Division of Translational Cancer Research, Department of Laboratory Medicine, Lund University, Lund, Sweden

**Keywords:** drug screening, neuroblastoma, patient-derived models, patient-derived xenograft, pediatric cancer, precision medicine, tumor organoids

## Abstract

Neuroblastoma is a childhood cancer derived from the sympathetic nervous system. High-risk neuroblastoma patients have a poor overall survival and account for ~15% of childhood cancer deaths. There is thus a need for clinically relevant and authentic models of neuroblastoma that closely resemble the human disease to further interrogate underlying mechanisms and to develop novel therapeutic strategies. Here we review recent developments in patient-derived neuroblastoma xenograft models and *in vitro* cultures. These models can be used to decipher mechanisms of metastasis and treatment resistance, for drug screening, and preclinical drug testing. Patient-derived neuroblastoma models may also provide useful information about clonal evolution, phenotypic plasticity, and cell states in relation to neuroblastoma progression. We summarize current opportunities for, but also barriers to, future model development and application. Integration of patient-derived models with patient data holds promise for the development of precision medicine treatment strategies for children with high-risk neuroblastoma.

## 1 Introduction

Despite significant academic, industrial, and clinical efforts, successfully translating preclinical findings to clinical trials and practice remains challenging (1–4) and less than 10% of drugs entering oncology clinical trials are eventually approved for clinical use (1, 5). Furthermore, these efforts and failures come at high financial and ethical costs. Pediatric malignancies have special considerations, since clinical drug testing is even more restricted by the relatively small number of patients and the ethics related to long-term side-effects in children. Involvement of multiple stakeholders is important to address the lack of childhood-specific drug development in the pharmaceutical sector (6, 7). Thus, there is an urgent need for clinically relevant and biologically accurate preclinical models to minimize these current bottlenecks to drug development and implementation (8).

Neuroblastoma (NB) is the most common solid extracranial pediatric tumor, accounting for ~15% of pediatric oncology deaths (9, 10). NB can be regarded as an aberration of neural crest development, and although it can arise anywhere along the sympathetic nervous system, most primary tumors are found in the adrenal gland (11). NB is biologically and clinically heterogenous, and patients are stratified into different risk groups based on tumor characteristics and disease presentation (12, 13). Clinical responses vary from spontaneous regression to metastatic and drug-resistant disease despite intensive treatment (13). Furthermore, patients often suffer from severe therapy-related long-term adverse effects (14).

NB is a copy number-driven disease with few targetable somatic mutations found at diagnosis, especially when compared with adult malignancies (15). The *MYCN* oncogene is amplified in ~20% of cases and is strongly correlated with aggressive phenotypes and unfavorable clinical outcomes (16). Other common chromosomal copy number changes, including 11q loss and 17q gain, are also poor prognostic features (17). Recurrent mutations are rare in NB but include *ALK* (9%), *ATRX* (7%), and *PTEN* (3%) mutations (15). In relapsed tumors, genome-wide sequencing has revealed a higher prevalence of recurrent mutations in targetable pathways, such as RAS-MAPK (18, 19), but at relatively low frequencies. Recent transcriptional and epigenetic analyses suggest that NB cells can adopt at least two phenotypic cell states, known as adrenergic (ADR)/differentiated and mesenchymal (MES)/immature (20–24). These findings highlight the heterogeneous and dynamic nature of NB.

Reliable, predictive, and authentic NB models are important because: (i) NB is uncommon, so sufficiently powered clinical trials are challenging and patient material is scarce for molecular studies (25), placing extra weight on the translatability of preclinical results; (ii) preclinical models that accurately recapitulate known clinical, genetic, and transcriptional intra- and inter-tumor heterogeneity are important for the identification of effective therapeutic targets; and (iii) patient-derived (PD) models resemble the clinical scenario better than conventional models and can therefore be used to screen for and test the most promising and safe novel therapies.

**Figure 1:**
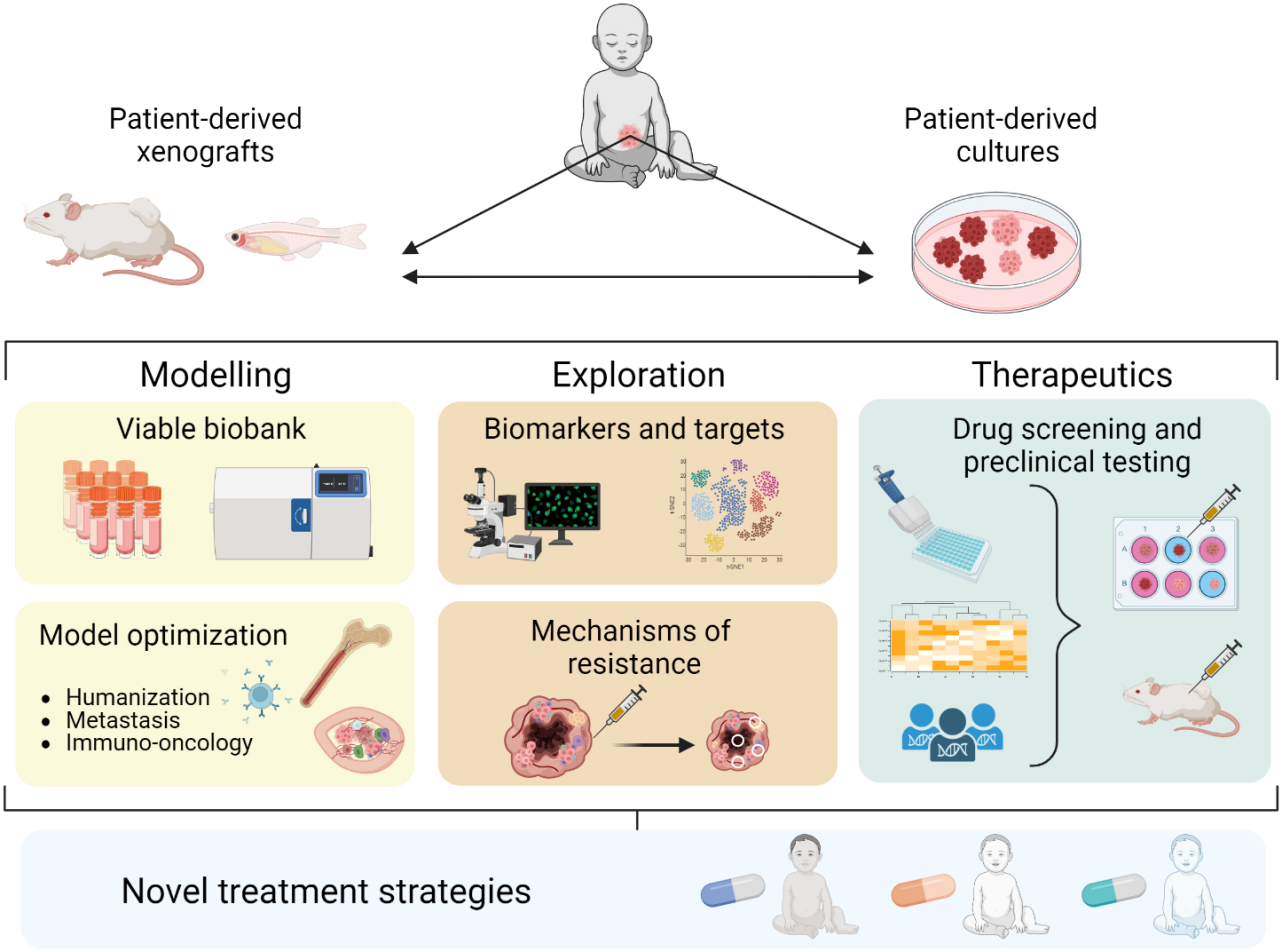
Establishment and application of PD models in NB contributing to novel treatment strategies.

Here we discuss recent progress in patient-derived xenografts (PDXs) and PD *in vitro* cultures as preclinical NB models. We summarize the development of different PD models, their utility in studying biological mechanisms and treatment responses, and how they can be utilized for preclinical drug testing to improve treatment strategies against NB (**Figure 1**).

## 2 PD NB models

Conventional cell lines have been used as laboratory models for decades, and they have provided valuable knowledge about tumor biology and drug efficacy in many cancer types. However, cancer cell lines are usually passaged under serum-containing conditions for years and thus their molecular profiles often differ from the original patient tumor (26, 27). This matters in terms of model fidelity, especially when considering clinical applicability; for example, clinically important features such as drug resistance might be lost after long-term *in vitro* passaging (27). PD NB models are established directly from tumor material obtained from children after parental informed consent. PD models have been shown to better reflect the features (e.g., treatment response) of their original tumors, compared with conventional models (28–31). PDXs have now been established from many diverse tumor types of adult and pediatric cancers including NB (32–35). Over the last few decades, the cancer research community has gradually turned towards PD model systems (8), and the US National Cancer Institute recently decided to replace its panel of human cancer cell lines (NCI-60) with well-characterized PDX models (36) for drug screening.

### 2.1 Establishment of NB PDXs *in vivo*

NB PDXs have been established in immunocompromised mice, mainly by implanting tumor samples or cells obtained from patients next to the adrenal gland (orthotopic implantation) or subcutaneously (ectopic/heterologous implantation). Established orthotopic PDX tumors can be monitored by clinical imaging techniques such as FDG-PET or MRI (37), and they have been shown to retain important patient tumor characteristics such as invasive growth patterns into surrounding tissues and spontaneous metastatic capacity to the bone marrow, lungs, and liver (37–39). PDX models retain NB-specific molecular features, including cellular differentiation status, protein marker expression (synaptophysin, chromogranin A, NCAM/CD56), chromosomal copy number changes (including 1p loss, *MYCN* amplification, 17q gain), mutational profiles, and DNA methylation status (32, 34, 37–41). Transcriptional analysis of orthotopic NB PDXs has shown that they also retain a certain degree of patient-specific gene expression, indicating transcriptional stability, from the corresponding NBs (32, 39, 40). Thus, although a PDX is established from only a fragment of the original patient tumor, data from multiple laboratories have shown that NB PDXs represent the main and clinically relevant features of NB patient tumors. There are now several sources of NB PDX tumors (detailed in (42)), including the US Pediatric Preclinical *In Vivo* Testing Consortium (PIVOT) and the European ITCC-P4 - Pediatric Preclinical Proof of Concept Platform.

The site of implantation affects the PDX model: orthotopic implantation has a higher engraftment rate and tumors grow faster than those implanted subcutaneously (32, 41). The human tumor microenvironment (TME) is gradually lost *in vivo*. Instead, orthotopic NB PDXs have been shown to contain a murine TME including for example vascularization, pericytes, macrophages, and extracellular matrix resembling the architecture in the parental NB (38). Potential functional differences between human and mouse TMEs are not fully elucidated and this uncertainty is important to consider (43). It has been debated whether the use of mice as hosts leads to murine-specific tumor evolution during PDX engraftment and propagation (44, 45). However, serial *in vivo* passaging of orthotopic NB PDXs for up to two years has shown that PDXs retain key genetic aberrations (e.g., 1p loss, *MYCN* amplification, and 17q gain) and acquire only minor genetic changes over time, as would be expected from their natural evolution (39). Clonal dynamics studies during tumor progression in PDXs have shown the presence of branched evolution, clonal sweeps, and convergent evolution of specific small deletions in potentially tumor-associated genes (46), a pattern similar to tumor evolution in NB patients (47).

There have been cases where human lymphomas have developed at the site of NB-cell injection (41, 48), or when murine-derived tumors have replaced the human PDX (49, 50), so thorough and frequent characterization of PDXs is necessary. Setting up robust biobanks for storage of well-characterized, early passage PDX-tumors will be of great benefit to the research community (8).

While most PDX models have been established in mice, zebrafish are increasingly used as hosts for implantation of PD tumor cells, including NB. Zebrafish allow for rapid and low-cost preclinical drug screening in an intact organism that may inform about precision medicine strategies in NB (51, 52). Furthermore, genetically-modified strains are available and tumors can be visualized from an early stage and followed dynamically. However, challenges in translating drug testing findings to patients include limited toxicity and pharmacodynamic data (51), temperature differences, and the non-mammalian TME.

PD cells and tumor biopsies have also been implanted into chick embryos with a high engraftment rate, forming metastases only from tumor cells from patients with metastatic NB and not from localized disease (53). NB cells migrated along the embryonic aorta and along peripheral nerves, demonstrating these as major routes for metastatic dissemination. This model allows for investigation of tumor progression and metastasis in an embryonic environment *in vivo* (53). However, the clinical relevance of the models remains uncertain.

### 2.2 PD cultures *in vitro*

PD tumor cultures are established *in vitro* directly from patients and can be grown as tumor organoids (PDOs), spheroids, or as semi-attached or attached cultures. PD cultures provide an opportunity to test potential therapeutics in a faster, high-throughput manner compared to PDXs.

PD NB cultures are isolated directly from primary or metastatic tumors from patients and are cultured in serum-free medium with defined growth factors to avoid neurospecific differentiation. This is best achieved on low-attachment plastics, with or without Matrigel or other scaffolding materials. Several groups have shown that PD NB cultures retain the copy-number profiles, mutation patterns, and other genetic and phenotypic characteristics of the tumor of origin (54–57) in both Matrigel and as free-floating spheres, but PDOs in Matrigel have better self-organization (56). Establishing PD cultures from different stages and subgroups of NB has been challenging. In general, more aggressive, *MYCN*-amplified, and metastatic tumors are easier to propagate *in vitro*. Recent advances in 3D scaffolding with hydrogels and porous scaffolding (reviewed in (58)) together with further optimization of culture conditions might increase the probability of successful establishment. Characterization of culture conditions is also important to understand how different transcriptional cell states might be maintained *in vitro*. Notably, it is important to verify NB identity and lack of contamination with other cells, which can otherwise overtake PD NB cultures (59, 60).

Since the limited number of NB patients restricts the number of models, a complementary approach is to use PDX-derived *in vitro* cultures after expansion of patient material *in vivo* (37, 40, 57, 61–63). Similar to PD cultures, PDX-derived NB cells can be grown adherent or as free-floating 3D cultures, and they retain patient-specific genomic aberrations as well as tumorigenic and metastatic capacity *in vivo* (62). Drug responses between NB PD- and PDX-derived cultures are highly correlated, suggesting that these models can be used interchangeably for drug testing (41). Biobanking of PD- and PDX-derived NB cultures will be a very important tool for future drug screening and larger preclinical drug testing (8).

## 3 Applications of PD NB models

Conventional cell lines, cell-line derived xenografts, and genetically engineered mouse models have been the main preclinical tools used to study resistance mechanisms and for drug testing. By using PD models that retain the main characteristics and heterogeneity of the original tumors, patient tumors and their treatment response can be better represented in the laboratory, thereby bridging the gap between preclinical models and the clinic.

### 3.1 Identification of treatment resistance mechanisms and biomarkers

Treatment resistance, relapse after therapy, and metastasis are urgent clinical problems in NB. A few studies have used PD models to identify diverse mechanisms implicated in NB invasion, migration, metastasis (64, 65), and resistance to specific chemotherapies (66). Using a clinically relevant treatment protocol (COJEC-induction therapy), NB PDXs show similar chemotherapy responses to their corresponding patients, suggesting that NB PDXs are useful for modelling chemoresistance and relapse (46). The models showed that chemoresistant NBs have a lower ADR signature and enrichment for an immature MES-like phenotype, suggesting an association between the MES cell state and relapse (46). These results are consistent with recent findings in the clinical setting (24, 67). The ability to accurately model treatment responses and their association with phenotypic cell states make *in vivo* PDXs a very promising tool to explore NB phenotypes in a reproducible manner, as well as characterizing the role of phenotypic plasticity in acquired and intrinsic resistance.

Reliable biomarkers for monitoring tumor responses are important for longer-term studies of relapse and resistance, and in clinical diagnostics. NB PDXs reproduce the patient’s relative levels of circulating metanephrines (68). Given that metanephrines are tumor progression biomarkers [plasma levels correlate with tumor volume (69)], this could pave the way for a minimally invasive method of monitoring tumor response/resistance in orthotopic PDX models. Another approach for monitoring responses is with gene signatures as recently optimized and used in a therapeutic study for high-risk relapsed NB in PD models (70).

### 3.2 Drug testing

#### 3.2.1 Application in the preclinical setting

Preclinical PD model testing is now highly recommended for proof-of-concept studies of new drugs and drug combinations aiming for clinical trials in the pediatric population (8). Many NB targets identified in patients have been tested in PD models *in vitro* and *in vivo*, allowing the evaluation of specific responses in tumors harboring different underlying, molecular alterations. Some known genetic vulnerabilities in NB are still under investigation, while others, for example ALK, have been clinically tested (71, 72). **Table 1** presents an overview of recent preclinical drug investigations of established and novel NB targets that were identified and tested in PD models.

**Table 1:** Selected preclinical drug testing studies using NB patient-derived models.

Target	Description	PD *in vivo*	PD *in vitro*	References
Small molecules				
CNR2, MAPK8	TargetTranslator tool for drug discovery	✓	✓	Almstedt et al., 2020 (52)
SHP2	Targeting tumors with low expression of NF1	✓	✓	Cai et al., 2022 (73)
ROS (ferroptosis)	Antioxidant pathways inhibition	✓	✓	Floros et al., 2021 (74)
PIM/PI3K/mTOR	New triple inhibitor	✓	✓	Mohlin et al., 2019 (75)
RAS/antimitotic	Antimitotic effects of rigosertib	✓	✓	Radke et al., 2021 (76)
KSP (Eg5)	HTS identifying new inhibitors, complete response in PDXs	✓	✓	Hansson et al., 2020 (77)
KSP (Eg5)	New oral inhibitor, liver metastasis model	✓	–	Masanas et al., 2020 (78)
Antimitotic	New inhibitor in taxane- and chemoresistant models	✓	–	Grohman et al., 2021 (79)
PARP, ATM	Targeting DNA damage, ATRX mutant NB	✓	–	George et al., 2020 (80)
PP2A	New PP2A activators	✓	–	Bownes et al., 2022 (81)
TOP2B	HTS, redefining MoA of an inhibitor	✓	–	Pan et al., 2021 (82)
CHK1	Prexasertib with chemotherapy in NB	✓	–	Lowery et al., 2019 (83)
ALK, TRK, JAK2/STAT, Src/FAK	Multikinase targeting	✓	–	O´Donohue et al., 2021 (84)
PHGDH	LC-MS-based proteomics, MYCN-associated targets	✓	–	Arlt et al., 2021 (85)
PGDB5	DNA transposase inhibition impairs DNA repair	✓	–	Henssen et al., 2017 (86)
ALK	New molecule: lorlatinib	✓	–	Infarinato et al., 2016 (87)
CAIX/CAXII	New inhibitor, organotypic slice culture	–	✓	Huo et al., 2022 (88)
Drug combinations				
CHK1+RRM2	Synergistic effects on replication stress	✓	✓	Nunes et al., 2022 (89)
ALK+chemo	Crizotinib combination with chemotherapy	✓	✓	Krytska et al., 2016 (90)
ALK+CDK4/6	Combination screen identifying new synergistic targets	✓	✓	Wood et al., 2017 (91)
ALK+PIM1	CRISPR screen, targeting ALK resistance	✓	✓	Trigg et al., 2019 (92)
BCL2+MDM2; BCL2+ CYCLO/TOPO; BCL2+MCL1	Venetoclax in combinations with clinically relevant agents	✓	✓	Dalton et al., 2021 (93)
BCL2+ferentidine	Synergistic effects of venetoclax and ferentidine	✓	✓	Nguyen et al., 2019 (94)
MGMT+TMZ+TOP2;	Combination of drugs targeting DNA damage	✓	✓	Hindle et al., 2021 (95)
HDAC+DOXO	Rapid zebra fish screen	✓	✓	Wrobel et al., 2020 (96)
PLK1/BRD4	Screen of dual inhibitors	✓	–	Timme et al., 2020 (97)
BCL-2+Aurora A	Screen of new venetoclax combinations	✓	–	Ham et al., 2016 (98)
TBX2	TF addiction is targeted by BETi + CDK7i	–	✓	Decaester et al., 2018 (99)
Immunotherapy & other				
ALK	Antibody-toxin conjugate directed towards ALK	✓	✓	Sano et al., 2019 (100)
Oncolytic therapeutics	oHSV expressing mIL-12	✓	✓	Quinn et al., 2022 (101)
aNK cells+anti-GD2	Residual disease targeting	✓	–	Barry et al., 2019 (102)
IL-15+anti-GD2	Substitution of IL15 for IL2 to limit toxicities	✓	–	Nguyen et al., 2019 (103)
IL-15/21+anti-GD2	GD2-targeted IL delivery in orthotopic models	✓	–	Nguyen et al., 2022 (104)
TOP1+anti-GD2	GD2-targeted nanoparticle delivery of SN-38	✓	–	Monderrubio et al., 2017 (105)

ALK, anaplastic lymphoma kinase; ATM, ataxia-telangiectasia mutated serine/threonine kinase; BCL, B-cell lymphoma; BET, bromodomain and extra-terminal domain; BRD4, bromodomain containing 4; CA, carbonic anhydrase; CDK, cyclin-dependent kinase; CHK, checkpoint kinase; CNR, cannabinoid receptor; CYCLO, cyclophosphamide; DOXO, doxycycline; FAK, focal adhesion kinase; GD2, disialoganglioside; HDAC, histone deacetylase; IL, interleukin; JAK, Janus kinase; KSP, kinesin spindle protein; MAPK, mitogen-activated protein kinase; MCL, induced myeloid leukemia cell differentiation; MDM, E3 ubiquitin-protein ligase; MGMT, O-6-methylguanine-DNA methyltransferase; oHSV, oncolytic herpes simplex virus; mTOR, mammalian target of rapamycin; PARP, poly (ADP-ribose) polymerase; PGBD5, PiggyBac transposable element derived 5; PHGDH, phosphoglycerate dehydrogenase; PI3K, phosphoinositide 3-kinases; PIM, Pim-1 proto-oncogene, serine/threonine kinase; PLK, polo-like kinase; PP2A, protein phosphatase 2; ROS, reactive oxygen species; RRM2, ribonucleotide reductase regulatory subunit M2; SHP2, protein tyrosine phosphatase; STAT, signal transducer and activator of transcription; TBX, T-box transcription factor; TF, transcription factor; TOP2, topoisomerase II alpha; TOPO, topotecan; TRK, tropomyosin receptor kinase.

High-throughput screens (HTS) *in vitro* can facilitate the discovery of specific targets and/or drugs using CRISPR/siRNA or phenotypic response. Most screening approaches still use conventional cell lines, but more recently PDX-derived cultures of high-risk NB have been used for the initial identification, for example for a KSP inhibitor (77). Compounds identified in drug screens can be further verified *in vivo* in PDXs.

In our experience, PD models show high intra-model variability in drug response (46, 75, 77) and are often less responsive to different treatments than conventional cell lines and xenografts [discussed also in (29)]. The lower sensitivity of PD models could indicate an even smaller effect in patients, thus providing important information with respect to optimal clinical implementation.

#### 3.2.2 Application for precision medicine in the clinic

The possibility of identifying actionable genetic alterations in pediatric cancers has contributed to optimism that the approach is useful for clinical trial design and target identification for high-risk and relapsed pediatric tumors, including high-risk NB (71, 72). Langenberg et al. thoroughly summarized current pediatric precision medicine programs around the world (106). Many of the programs/consortia [Pediatric MATCH (US) or INFORM (Europe)] have enabled patients to receive treatments tailored to the individual tumor’s molecular profile (107–109). However, relatively few identified mutations (<30%) have led to targeted therapies (106, 107, 110). This highlights the need for molecular profiling of patients to be backed up by real-time functional testing of drug sensitivities in PD models.

Both the PIVOT (US, earlier PPTC) and ITCC-P4 (Europe) repositories hold PDXs. Considering that PDXs take time to establish, co-clinical avatar studies are generally very difficult. Nevertheless, the rarity of pediatric cancers and scarcity of models representing specific subtypes within pediatric tumors makes those repositories a valuable resource for the accelerated development and translation of novel therapeutics into early phase trials (34, 111, 112). Lau et al. developed a pediatric precision medicine platform (including a few high-risk NBs) of PDX models and HTS in PDOs, observing a correlation between PDX results, HTS-PDOs, and the clinical responses in patients (113). Importantly, the addition of functional drug testing to a genome-only analysis increased the number of patients with drug options by also identifying drug sensitivities not associated with molecular hallmarks (113).

Real-time drug testing for the immediate benefit of the patient is likely to be more feasible in PDO models where the time for establishment is much shorter and the readout can be performed with a higher throughput. The COMPASS consortium (Clinical implementation Of Multidimensional PhenotypicAl drug SenSitivities in paediatric precision oncology) is a large-scale effort to implement HTS in PD models. This European collaborative platform aims to implement PDO screening for individualized drug sensitivity assessment and therapy (114). Recently, the network also standardized drug scoring tools and developed machine learning approaches (115, 116).

## 4 Current and future model optimization

Although the successful establishment of PD NB models is encouraging, certain aspects can still be improved. For example, the distribution and function of the extracellular matrix (ECM) has been shown to influence NB progression in patient samples (117, 118). Consistently, modulation of ECM components induces specific cell behaviors of PD NB cultures (63). Optimization of ECM conditions could thus contribute to improved NB modelling.

The lack of a complete immune system is a limitation of most PDX models, since PD tumors are generally implanted in immunocompromised mouse strains (e.g., NSG) to permit tumor engraftment. Reconstitution of a humanized immune system, for example by injection of human hematopoietic stem cells into sub-lethally irradiated mice, could improve the immune status of the models (119). A technically advanced humanized mouse strain (MISTRG) supports the intrinsic development of human natural killer (NK) cells after bone marrow transplantation (120). When combined with orthotopic NB PDXs, these mice have allowed the identification of immune modulating functions in common between PDXs and patient tumors and suggest that the model is useful for immuno-oncology studies in general and in NB in particular (121). The use of PDO and stromal/immune cell co-cultures can be applied *in vitro*, where it has been very challenging to optimize culture conditions for multiple cell types over longer periods (122, 123). Co-cultures of NB organoids and peripheral blood mononuclear cells (from a healthy donor) were recently used to test a novel immunotherapy (124).

Innovative technological advances have suggested that microfluidics (lab-on-a-chip) and bioprinting may provide future systems for studying tumor cell and stromal/immune cell interactions. Functional short-time cultures of both tumor cells and immune cells in a microfluidic system have been reported for adult cancers (125, 126) and might be applicable also to pediatric cancers. Very recently, the first bioprinted, vascularized NB microenvironment on a fluidic chip was reported (127). Implantation of cell line-derived NB spheroids led to NB cell survival for two weeks and successful micro-vessel infiltration of the spheroids (127). A different study managed to establish PD NB organotypic slice cultures that could potentially preserve an intact NB tumor microenvironment (88). This study used a perfusion-based bioreactor to force medium through the tissue, thereby providing continuous nutrient delivery to the whole tumor.

Orthotopic NB PDXs retain spontaneous metastatic capacity *in vivo* to the lungs, liver, and bone marrow, mimicking the entire process from primary tumor growth to invasion and metastasis (37, 39). However, the TME is generally murine (38) and there are uncertainties about cross reactivity between human NB cells and the mouse TME. The presence of human mesenchymal stem cells can increase growth and metastasis of NB cells *in vivo* (128), suggesting species preference. Recent advances in tissue engineering have produced *in vivo* models of humanized bone (so-called ossicles) in mice. Implanted PDX-derived NB cells form osteolytic tumor lesions in the ossicles and display higher and faster engraftment rates than in mouse bone (129). This model could thus be valuable for the investigation of human NB growth and treatment responses in a humanized metastatic niche.

Further optimization of PD models to account for more patient-like microenvironmental factors both *in vivo* and *in vitro* is ongoing and will likely contribute to improved translatability.

## 5 Conclusions

The use of clinically relevant preclinical models is of immense importance in childhood cancers, such as high-risk NB, where the access to patient material is limited. PD models reflect the characteristics of the tumor of origin better than conventional *in vivo* and *in vitro* models. Nevertheless, ongoing efforts may further optimize their translational relevance. Existing NB PDXs and PD cultures have been – and continue to be – used to decipher therapy resistance and for target identification and drug testing. Future studies will need to investigate how PD models can be used to exploit phenotypic plasticity and NB cell states in preclinical studies to better benefit NB patients.

## Author contributions

KA, KR, AA, AS, AM and DB wrote and reviewed the manuscript. KA and DB supervised the project. All authors contributed to the article and approved the submitted version.

## References

[1] Wong CH Siah KW Lo AW. Estimation of clinical trial success rates and related parameters. Biostatistics (2019) 20(2):273–86. doi: 10.1093/biostatistics/kxx069 PMC6409418 29394327

[2] Sharpless NE Depinho RA. The mighty mouse: Genetically engineered mouse models in cancer drug development. Nat Rev Drug discovery (2006) 5(9):741–54. doi: 10.1038/nrd2110 16915232

[3] Johnson JI Decker S Zaharevitz D Rubinstein LV Venditti JM Schepartz S. Relationships between drug activity in NCI preclinical *in vitro* and *in vivo* models and early clinical trials. Br J cancer (2001) 84(10):1424–31. doi: 10.1054/bjoc.2001.1796 PMC2363645 11355958

[4] Lin A Giuliano CJ Palladino A John KM Abramowicz C Yuan ML. Off-target toxicity is a common mechanism of action of cancer drugs undergoing clinical trials. Sci Transl Med (2019) 11(509):eaaw8412. doi: 10.1126/scitranslmed.aaw8412 31511426 PMC7717492

[5] Hay M Thomas DW Craighead JL Economides C Rosenthal J. Clinical development success rates for investigational drugs. Nat Biotechnol (2014) 32(1):40–51. doi: 10.1038/nbt.2786 24406927

[6] Moreno L Barone G DuBois SG Molenaar J Fischer M Schulte J. Accelerating drug development for neuroblastoma: Summary of the second neuroblastoma drug development strategy forum from innovative therapies for children with cancer and international society of paediatric oncology Europe neuroblastoma. Eur J Cancer (2020) 136:52–68. doi: 10.1016/j.ejca.2020.05.010 32653773

[7] Pearson AD Heenen D Kearns PR Goeres A Marshall LV Blanc P. 10-year report on the European paediatric regulation and its impact on new drugs for children's cancers. Lancet Oncol (2018) 19(3):285–7. doi: 10.1016/S1470-2045(18)30105-0 29508745

[8] Vassal G Houghton PJ Pfister SM Smith MA Caron HN Li XN. International consensus on minimum preclinical testing requirements for the development of innovative therapies for children and adolescents with cancer. Mol Cancer Ther (2021) 20(8):1462–8. doi: 10.1158/1535-7163.MCT-20-0394 34108262

[9] Gatta G Botta L Rossi S Aareleid T Bielska-Lasota M Clavel J. Childhood cancer survival in Europe 1999-2007: results of EUROCARE-5–a population-based study. Lancet Oncol (2014) 15(1):35–47. doi: 10.1016/S1470-2045(13)70548-5 24314616

[10] Park JR Eggert A Caron H. Neuroblastoma: biology, prognosis, and treatment. Hematol Oncol Clin North Am (2010) 24(1):65–86. doi: 10.1016/j.hoc.2009.11.011 20113896

[11] Matthay KK Maris JM Schleiermacher G Nakagawara A Mackall CL Diller L. Neuroblastoma. Nat Rev Dis Primers (2016) 2:16078. doi: 10.1038/nrdp.2016.78 27830764

[12] Pinto NR Applebaum MA Volchenboum SL Matthay KK London WB Ambros PF. Advances in risk classification and treatment strategies for neuroblastoma. J Clin Oncol Off J Am Soc Clin Oncol (2015) 33(27):3008–17. doi: 10.1200/JCO.2014.59.4648 PMC4567703 26304901

[13] Tolbert VP Matthay KK. Neuroblastoma: Clinical and biological approach to risk stratification and treatment. Cell Tissue Res (2018) 372(2):195–209. doi: 10.1007/s00441-018-2821-2 29572647 PMC5918153

[14] Cohen LE Gordon JH Popovsky EY Gunawardene S Duffey-Lind E Lehmann LE. Late effects in children treated with intensive multimodal therapy for high-risk neuroblastoma: High incidence of endocrine and growth problems. Bone Marrow Transplant (2014) 49(4):502–8. doi: 10.1038/bmt.2013.218 24442245

[15] Pugh TJ Morozova O Attiyeh EF Asgharzadeh S Wei JS Auclair D. The genetic landscape of high-risk neuroblastoma. Nat Genet (2013) 45(3):279–84. doi: 10.1038/ng.2529 PMC3682833 23334666

[16] Brodeur GM Seeger RC Schwab M Varmus HE Bishop JM. Amplification of n-myc in untreated human neuroblastomas correlates with advanced disease stage. Sci (New York NY) (1984) 224(4653):1121–4. doi: 10.1126/science.6719137 6719137

[17] Cohn SL Pearson AD London WB Monclair T Ambros PF Brodeur GM. The international neuroblastoma risk group (INRG) classification system: an INRG task force report. J Clin Oncol Off J Am Soc Clin Oncol (2009) 27(2):289–97. doi: 10.1200/JCO.2008.16.6785 PMC2650388 19047291

[18] Schramm A Koster J Assenov Y Althoff K Peifer M Mahlow E. Mutational dynamics between primary and relapse neuroblastomas. Nat Genet (2015) 47(8):872–7. doi: 10.1038/ng.3349 26121086

[19] Eleveld TF Oldridge DA Bernard V Koster J Daage LC Diskin SJ. Relapsed neuroblastomas show frequent RAS-MAPK pathway mutations. Nat Genet (2015) 47(8):864–71. doi: 10.1038/ng.3333 PMC4775079 26121087

[20] van Groningen T Koster J Valentijn LJ Zwijnenburg DA Akogul N Hasselt NE. Neuroblastoma is composed of two super-enhancer-associated differentiation states. Nat Genet (2017) 49(8):1261–6. doi: 10.1038/ng.3899 28650485

[21] Boeva V Louis-Brennetot C Peltier A Durand S Pierre-Eugène C Raynal V. Heterogeneity of neuroblastoma cell identity defined by transcriptional circuitries. Nat Genet (2017) 49(9):1408–13. doi: 10.1038/ng.3921 28740262

[22] Jansky S Sharma AK Körber V Quintero A Toprak UH Wecht EM. Single-cell transcriptomic analyses provide insights into the developmental origins of neuroblastoma. Nat Genet (2021) 53(5):683–93. doi: 10.1038/s41588-021-00806-1 33767450

[23] Bedoya-Reina OC Li W Arceo M Plescher M Bullova P Pui H. Single-nuclei transcriptomes from human adrenal gland reveal distinct cellular identities of low and high-risk neuroblastoma tumors. Nat Commun (2021) 12(1):5309. doi: 10.1038/s41467-021-24870-7 34493726 PMC8423786

[24] Gartlgruber M Sharma AK Quintero A Dreidax D Jansky S Park Y-G. Super enhancers define regulatory subtypes and cell identity in neuroblastoma. Nat Cancer (2021) 2(1):114–28. doi: 10.1038/s43018-020-00145-w 35121888

[25] Fletcher JI Ziegler DS Trahair TN Marshall GM Haber M Norris MD. Too many targets, not enough patients: Rethinking neuroblastoma clinical trials. Nat Rev Cancer (2018) 18(6):389–400. doi: 10.1038/s41568-018-0003-x 29632319

[26] Lee J Kotliarova S Kotliarov Y Li A Su Q Donin NM. Tumor stem cells derived from glioblastomas cultured in bFGF and EGF more closely mirror the phenotype and genotype of primary tumors than do serum-cultured cell lines. Cancer Cell (2006) 9(5):391–403. doi: 10.1016/j.ccr.2006.03.030 16697959

[27] Gillet JP Calcagno AM Varma S Marino M Green LJ Vora MI. Redefining the relevance of established cancer cell lines to the study of mechanisms of clinical anti-cancer drug resistance. Proc Natl Acad Sci United States America (2011) 108(46):18708–13. doi: 10.1073/pnas.1111840108 PMC3219108 22068913

[28] Hidalgo M Amant F Biankin AV Budinská E Byrne AT Caldas C. Patient-derived xenograft models: An emerging platform for translational cancer research. Cancer discovery (2014) 4(9):998–1013. doi: 10.1158/2159-8290.CD-14-0001 25185190 PMC4167608

[29] Gao H Korn JM Ferretti S Monahan JE Wang Y Singh M. High-throughput screening using patient-derived tumor xenografts to predict clinical trial drug response. Nat Med (2015) 21(11):1318–25. doi: 10.1038/nm.3954 26479923

[30] van de Wetering M Francies HE Francis JM Bounova G Iorio F Pronk A. Prospective derivation of a living organoid biobank of colorectal cancer patients. Cell (2015) 161(4):933–45. doi: 10.1016/j.cell.2015.03.053 PMC6428276 25957691

[31] Calandrini C Schutgens F Oka R Margaritis T Candelli T Mathijsen L. An organoid biobank for childhood kidney cancers that captures disease and tissue heterogeneity. Nat Commun (2020) 11(1):1310. doi: 10.1038/s41467-020-15155-6 32161258 PMC7066173

[32] Stewart E Federico SM Chen X Shelat AA Bradley C Gordon B. Orthotopic patient-derived xenografts of paediatric solid tumours. Nature (2017) 549(7670):96–100. doi: 10.1038/nature23647 28854174 PMC5659286

[33] Brabetz S Leary SES Gröbner SN Nakamoto MW Şeker-Cin H Girard EJ. A biobank of patient-derived pediatric brain tumor models. Nat Med (2018) 24(11):1752–61. doi: 10.1038/s41591-018-0207-3 30349086

[34] Rokita JL Rathi KS Cardenas MF Upton KA Jayaseelan J Cross KL. Genomic profiling of childhood tumor patient-derived xenograft models to enable rational clinical trial design. Cell Rep (2019) 29(6):1675–89.e9. doi: 10.1016/j.celrep.2019.09.071 31693904 PMC6880934

[35] Tucker ER George S Angelini P Bruna A Chesler L. The promise of patient-derived preclinical models to accelerate the implementation of personalised medicine for children with neuroblastoma. J Pers Med (2021) 11(4):248. doi: 10.3390/jpm11040248 33808071 PMC8065808

[36] Ledford H. US Cancer institute to overhaul tumour cell lines. Nature (2016) 530(7591):391. doi: 10.1038/nature.2016.19364 26911756

[37] Braekeveldt N Wigerup C Gisselsson D Mohlin S Merselius M Beckman S. Neuroblastoma patient-derived orthotopic xenografts retain metastatic patterns and geno- and phenotypes of patient tumours. Int J cancer (2015) 136(5):E252–61. doi: 10.1002/ijc.29217 PMC4299502 25220031

[38] Braekeveldt N Wigerup C Tadeo I Beckman S Sanden C Jonsson J. Neuroblastoma patient-derived orthotopic xenografts reflect the microenvironmental hallmarks of aggressive patient tumours. Cancer letters (2016) 375(2):384–9. doi: 10.1016/j.canlet.2016.02.046 27000989

[39] Braekeveldt N von Stedingk K Fransson S Martinez-Monleon A Lindgren D Axelson H. Patient-derived xenograft models reveal intratumor heterogeneity and temporal stability in neuroblastoma. Cancer Res (2018) 78(20):5958–69. doi: 10.1158/0008-5472.CAN-18-0527 30154149

[40] Stewart E Shelat A Bradley C Chen X Federico S Thiagarajan S. Development and characterization of a human orthotopic neuroblastoma xenograft. Dev Biol (2015) 407(2):344–55. doi: 10.1016/j.ydbio.2015.02.002 PMC4995597 25863122

[41] Kamili A Gifford AJ Li N Mayoh C Chow SO Failes TW. Accelerating development of high-risk neuroblastoma patient-derived xenograft models for preclinical testing and personalised therapy. Br J cancer (2020) 122(5):680–91. doi: 10.1038/s41416-019-0682-4 PMC7054410 31919402

[42] Ornell KJ Coburn JM. Developing preclinical models of neuroblastoma: Driving therapeutic testing. BMC BioMed Eng (2019) 1:33. doi: 10.1186/s42490-019-0034-8 32903387 PMC7422585

[43] Bleijs M van de Wetering M Clevers H Drost J. Xenograft and organoid model systems in cancer research. EMBO J (2019) 38(15):e101654. doi: 10.15252/embj.2019101654 31282586 PMC6670015

[44] Ben-David U Ha G Tseng Y-Y Greenwald NF Oh C Shih J. Patient-derived xenografts undergo mouse-specific tumor evolution. Nat Genet (2017) 49:1567. doi: 10.1038/ng.3967 28991255 PMC5659952

[45] Woo XY Giordano J Srivastava A Zhao Z-M Lloyd MW de Bruijn R. Conservation of copy number profiles during engraftment and passaging of patient-derived cancer xenografts. Nat Genet (2021) 53(1):86–99. doi: 10.1038/s41588-020-00750-6 33414553 PMC7808565

[46] Mañas A Aaltonen K Andersson N Hansson K Adamska A Seger A. Clinically relevant treatment of PDX models reveals patterns of neuroblastoma chemoresistance. Sci Adv (2022) 8(43):eabq4617. doi: 10.1016/j.celrep.2019.09.071 36306349 PMC9616506

[47] Karlsson J Valind A Holmquist Mengelbier L Bredin S Cornmark L Jansson C. Four evolutionary trajectories underlie genetic intratumoral variation in childhood cancer. Nat Genet (2018) 50(7):944–50. doi: 10.1038/s41588-018-0131-y 29867221

[48] Williams AP Stewart JE Stafman LL Aye JM Mroczek-Musulman E Ren C. Corruption of neuroblastoma patient derived xenografts with human T cell lymphoma. J Pediatr Surg (2019) 54(10):2117–9. doi: 10.1016/j.jpedsurg.2018.10.051 PMC6476711 30391152

[49] Moyer AM Yu J Sinnwell JP Dockter TJ Suman VJ Weinshilboum RM. Spontaneous murine tumors in the development of patient-derived xenografts: a potential pitfall. Oncotarget (2019) 10(39):3924–30. doi: 10.18632/oncotarget.27001 PMC6570466 31231469

[50] Tillman H Janke LJ Funk A Vogel P Rehg JE. Morphologic and immunohistochemical characterization of spontaneous Lymphoma/Leukemia in NSG mice. Vet Pathol (2020) 57(1):160–71. doi: 10.1177/0300985819882631 PMC8686194 31736441

[51] Gatzweiler C Ridinger J Herter S Gerloff XF ElHarouni D Berker Y. Functional therapeutic target validation using pediatric zebrafish xenograft models. Cancers (2022) 14(3):849. doi: 10.3390/cancers14030849 35159116 PMC8834194

[52] Almstedt E Elgendy R Hekmati N Rosen E Warn C Olsen TK. Integrative discovery of treatments for high-risk neuroblastoma. Nat Commun (2020) 11(1):71. doi: 10.1038/s41467-019-13817-8 31900415 PMC6941971

[53] Delloye-Bourgeois C Bertin L Thoinet K Jarrosson L Kindbeiter K Buffet T. Microenvironment-driven shift of Cohesion/Detachment balance within tumors induces a switch toward metastasis in neuroblastoma. Cancer Cell (2017) 32(4):427–43.e8. doi: 10.1016/j.ccell.2017.09.006 29017055

[54] Bate-Eya LT Ebus ME Koster J den Hartog IJ Zwijnenburg DA Schild L. Newly-derived neuroblastoma cell lines propagated in serum-free media recapitulate the genotype and phenotype of primary neuroblastoma tumours. Eur J Cancer (2014) 50(3):628–37. doi: 10.1016/j.ejca.2013.11.015 24321263

[55] Barton J Pacey K Jain N Kasia T Edwards D Thevanesan C. Establishment and phenotyping of neurosphere cultures from primary neuroblastoma samples. F1000Res (2019) 8:823. doi: 10.12688/f1000research.18209.1 31316758 PMC6611133

[56] Fusco P Parisatto B Rampazzo E Persano L Frasson C Di Meglio A. Patient-derived organoids (PDOs) as a novel *in vitro* model for neuroblastoma tumours. BMC cancer (2019) 19(1):970. doi: 10.1186/s12885-019-6149-4 31638925 PMC6802324

[57] Thole TM Toedling J Sprüssel A Pfeil S Savelyeva L Capper D. Reflection of neuroblastoma intratumor heterogeneity in the new OHC-NB1 disease model. Int J cancer (2020) 146(4):1031–41. doi: 10.1002/ijc.32572 31304977

[58] Nolan JC Frawley T Tighe J Soh H Curtin C Piskareva O. Preclinical models for neuroblastoma: Advances and challenges. Cancer letters (2020) 474:53–62. doi: 10.1016/j.canlet.2020.01.015 31962141

[59] Hansford LM McKee AE Zhang L George RE Gerstle JT Thorner PS. Neuroblastoma cells isolated from bone marrow metastases contain a naturally enriched tumor-initiating cell. Cancer Res (2007) 67(23):11234–43. doi: 10.1158/0008-5472.CAN-07-0718 18056449

[60] Mohlin S Pietras A Wigerup C Ora I Andang M Nilsson K. Tumor-initiating cells in childhood neuroblastoma–letter. Cancer Res (2012) 72(3):821–2. author reply 3. doi: 10.1158/0008-5472.CAN-11-1761 22298597

[61] Coulon A Flahaut M Mühlethaler-Mottet A Meier R Liberman J Balmas-Bourloud K. Functional sphere profiling reveals the complexity of neuroblastoma tumor-initiating cell model. Neoplasia (2011) 13(10):991–1004. doi: 10.1593/neo.11800 22028624 PMC3201575

[62] Persson CU von Stedingk K Bexell D Merselius M Braekeveldt N Gisselsson D. Neuroblastoma patient-derived xenograft cells cultured in stem-cell promoting medium retain tumorigenic and metastatic capacities but differentiate in serum. Sci Rep (2017) 7(1):10274. doi: 10.1038/s41598-017-09662-8 28860499 PMC5579187

[63] Gavin C Geerts N Cavanagh B Haynes M Reynolds CP Loessner D. Neuroblastoma invasion strategies are regulated by the extracellular matrix. Cancers (2021) 13(4):736. doi: 10.3390/cancers13040736 33578855 PMC7916632

[64] Ben Amar D Thoinet K Villalard B Imbaud O Costechareyre C Jarrosson L. Environmental cues from neural crest derivatives act as metastatic triggers in an embryonic neuroblastoma model. Nat Commun (2022) 13(1):2549. doi: 10.1038/s41467-022-30237-3 35538114 PMC9091272

[65] Bownes LV Williams AP Marayati R Stafman LL Markert H Quinn CH. EZH2 inhibition decreases neuroblastoma proliferation and *in vivo* tumor growth. PloS One (2021) 16(3):e0246244. doi: 10.1371/journal.pone.0246244 33690617 PMC7942994

[66] Koneru B Farooqi A Nguyen TH Chen WH Hindle A Eslinger C. ALT neuroblastoma chemoresistance due to telomere dysfunction-induced ATM activation is reversible with ATM inhibitor AZD0156. Sci Transl Med (2021) 13(607):eabd5750. doi: 10.1126/scitranslmed.abd5750 34408079 PMC9208664

[67] Sengupta S Das S Crespo AC Cornel AM Patel AG Mahadevan NR. Mesenchymal and adrenergic cell lineage states in neuroblastoma possess distinct immunogenic phenotypes. Nat Cancer (2022) 3(10):1228–46. doi: 10.1038/s43018-022-00427-5 PMC10171398 36138189

[68] Abid K Popovic MB Bourloud KB Schoumans J Grand-Guillaume J Grouzmann E. The noradrenergic profile of plasma metanephrine in neuroblastoma patients is reproduced in xenograft mice models and arise from PNMT downregulation. Oncotarget (2021) 12(1):49–60. doi: 10.18632/oncotarget.27858 33456713 PMC7800772

[69] Eisenhofer G Lenders JW Goldstein DS Mannelli M Csako G Walther MM. Pheochromocytoma catecholamine phenotypes and prediction of tumor size and location by use of plasma free metanephrines. Clin Chem (2005) 51(4):735–44. doi: 10.1373/clinchem.2004.045484 15718487

[70] Müller M Rösch L Najafi S Gatzweiler C Ridinger J Gerloff XF. Combining APR-246 and HDAC-inhibitors: A novel targeted treatment option for neuroblastoma. Cancers (2021) 13(17):4476. doi: 10.3390/cancers13174476 34503286 PMC8431508

[71] Mosse YP Fox E Teachey DT Reid JM Safgren SL Carol H. A phase II study of alisertib in children with Recurrent/Refractory solid tumors or leukemia: Children's oncology group phase I and pilot consortium (ADVL0921). Clin Cancer Res an Off J Am Assoc Cancer Res (2019) 25(11):3229–38. doi: 10.1158/1078-0432.CCR-18-2675 PMC6897379 30777875

[72] Mossé YP Lim MS Voss SD Wilner K Ruffner K Laliberte J. Safety and activity of crizotinib for paediatric patients with refractory solid tumours or anaplastic large-cell lymphoma: A children's oncology group phase 1 consortium study. Lancet Oncol (2013) 14(6):472–80. doi: 10.1016/S1470-2045(13)70095-0 PMC3730818 23598171

[73] Cai J Jacob S Kurupi R Dalton KM Coon C Greninger P. High-risk neuroblastoma with NF1 loss of function is targetable using SHP2 inhibition. Cell Rep (2022) 40(4):111095. doi: 10.1016/j.celrep.2022.111095 35905710 PMC10353975

[74] Floros KV Cai J Jacob S Kurupi R Fairchild CK Shende M. MYCN-amplified neuroblastoma is addicted to iron and vulnerable to inhibition of the system xc-/Glutathione axis. Cancer Res (2021) 81(7):1896–908. doi: 10.1158/0008-5472.CAN-20-1641 PMC9281612 33483374

[75] Mohlin S Hansson K Radke K Martinez S Blanco-Apiricio C Garcia-Ruiz C. Anti-tumor effects of PIM/PI3K/mTOR triple kinase inhibitor IBL-302 in neuroblastoma. EMBO Mol Med (2019) 11(8):e10058. doi: 10.15252/emmm.201810058 31310053 PMC6685085

[76] Radke K Hansson K Sjölund J Wolska M Karlsson J Esfandyari J. Anti-tumor effects of rigosertib in high-risk neuroblastoma. Transl Oncol (2021) 14(8):101149. doi: 10.1016/j.tranon.2021.101149 34118691 PMC8207190

[77] Hansson K Radke K Aaltonen K Saarela J Mañas A Sjölund J. Therapeutic targeting of KSP in preclinical models of high-risk neuroblastoma. Sci Transl Med (2020) 12(562):eaba4434. doi: 10.1126/scitranslmed.aba4434 32967973

[78] Masanas M Masiá N Suárez-Cabrera L Olivan M Soriano A Majem B. The oral KIF11 inhibitor 4SC-205 exhibits antitumor activity and potentiates standard and targeted therapies in primary and metastatic neuroblastoma models. Clin Transl Med (2021) 11(10):e533. doi: 10.1002/ctm2.533 34709738 PMC8516339

[79] Grohmann C Walker F Devlin M Luo MX Chüeh AC Doherty J. Preclinical small molecule WEHI-7326 overcomes drug resistance and elicits response in patient-derived xenograft models of human treatment-refractory tumors. Cell Death disease (2021) 12(3):268. doi: 10.1038/s41419-020-03269-0 33712556 PMC7955127

[80] George SL Lorenzi F King D Hartlieb S Campbell J Pemberton H. Therapeutic vulnerabilities in the DNA damage response for the treatment of ATRX mutant neuroblastoma. EBioMedicine (2020) 59:102971. doi: 10.1016/j.ebiom.2020.102971 32846370 PMC7452577

[81] Bownes LV Marayati R Quinn CH Beierle AM Hutchins SC Julson JR. Pre-clinical study evaluating novel protein phosphatase 2A activators as therapeutics for neuroblastoma. Cancers (2022) 14(8):1952. doi: 10.3390/cancers14081952 35454859 PMC9026148

[82] Pan M Wright WC Chapple RH Zubair A Sandhu M Batchelder JE. The chemotherapeutic CX-5461 primarily targets TOP2B and exhibits selective activity in high-risk neuroblastoma. Nat Commun (2021) 12(1):6468. doi: 10.1038/s41467-021-26640-x 34753908 PMC8578635

[83] Lowery CD Dowless M Renschler M Blosser W VanWye AB Stephens JR. Broad spectrum activity of the checkpoint kinase 1 inhibitor prexasertib as a single agent or chemopotentiator across a range of preclinical pediatric tumor models. Clin Cancer Res an Off J Am Assoc Cancer Res (2019) 25(7):2278–89. doi: 10.1158/1078-0432.CCR-18-2728 PMC6445779 30563935

[84] O'Donohue TJ Ibáñez G Coutinho DF Mauguen A Siddiquee A Rosales N. Translational strategies for repotrectinib in neuroblastoma. Mol Cancer Ther (2021) 20(11):2189–97. doi: 10.1158/1535-7163.MCT-21-0126 PMC8571000 34482287

[85] Arlt B Zasada C Baum K Wuenschel J Mastrobuoni G Lodrini M. Inhibiting phosphoglycerate dehydrogenase counteracts chemotherapeutic efficacy against MYCN-amplified neuroblastoma. Int J cancer (2021) 148(5):1219–32. doi: 10.1002/ijc.33423 33284994

[86] Henssen AG Reed C Jiang E Garcia HD von Stebut J MacArthur IC. Therapeutic targeting of PGBD5-induced DNA repair dependency in pediatric solid tumors. Sci Transl Med (2017) 9(414):eaam9078. doi: 10.1126/scitranslmed.aam9078 29093183 PMC5683417

[87] Infarinato NR Park JH Krytska K Ryles HT Sano R Szigety KM. The ALK/ROS1 inhibitor PF-06463922 overcomes primary resistance to crizotinib in ALK-driven neuroblastoma. Cancer discovery (2016) 6(1):96–107. doi: 10.1158/2159-8290.CD-15-1056 26554404 PMC4707106

[88] Huo Z Bilang R Supuran CT von der Weid N Bruder E Holland-Cunz S. Perfusion-based bioreactor culture and isothermal microcalorimetry for preclinical drug testing with the carbonic anhydrase inhibitor SLC-0111 in patient-derived neuroblastoma. Int J Mol Sci (2022) 23(6):3128. doi: 10.3390/ijms23063128 35328549 PMC8955558

[89] Nunes C Depestel L Mus L Keller KM Delhaye L Louwagie A. RRM2 enhances MYCN-driven neuroblastoma formation and acts as a synergistic target with CHK1 inhibition. Sci Adv (2022) 8(28):eabn1382. doi: 10.1126/sciadv.abn1382 35857500 PMC9278860

[90] Krytska K Ryles HT Sano R Raman P Infarinato NR Hansel TD. Crizotinib synergizes with chemotherapy in preclinical models of neuroblastoma. Clin Cancer Res an Off J Am Assoc Cancer Res (2016) 22(4):948–60. doi: 10.1158/1078-0432.CCR-15-0379 PMC4755925 26438783

[91] Wood AC Krytska K Ryles HT Infarinato NR Sano R Hansel TD. Dual ALK and CDK4/6 inhibition demonstrates synergy against neuroblastoma. Clin Cancer Res an Off J Am Assoc Cancer Res (2017) 23(11):2856–68. doi: 10.1158/1078-0432.CCR-16-1114 PMC5457336 27986745

[92] Trigg RM Lee LC Prokoph N Jahangiri L Reynolds CP Amos Burke GA. The targetable kinase PIM1 drives ALK inhibitor resistance in high-risk neuroblastoma independent of MYCN status. Nat Commun (2019) 10(1):5428. doi: 10.1038/s41467-019-13315-x 31780656 PMC6883072

[93] Dalton KM Krytska K Lochmann TL Sano R Casey C D'Aulerio A. Venetoclax-based rational combinations are effective in models of MYCN-amplified neuroblastoma. Mol Cancer Ther (2021) 20(8):1400–11. doi: 10.1158/1535-7163.MCT-20-0710 PMC10350345 34088831

[94] Nguyen TH Koneru B Wei SJ Chen WH Makena MR Urias E. Fenretinide *via* NOXA induction, enhanced activity of the BCL-2 inhibitor venetoclax in high BCL-2-Expressing neuroblastoma preclinical models. Mol Cancer Ther (2019) 18(12):2270–82. doi: 10.1158/1535-7163.MCT-19-0385 PMC9255909 31484706

[95] Hindle A Koneru B Makena MR Lopez-Barcons L Chen WH Nguyen TH. The O6-methyguanine-DNA methyltransferase inhibitor O6-benzylguanine enhanced activity of temozolomide + irinotecan against models of high-risk neuroblastoma. Anticancer Drugs (2021) 32(3):233–47. doi: 10.1097/CAD.0000000000001020 PMC9255907 33323683

[96] Wrobel JK Najafi S Ayhan S Gatzweiler C Krunic D Ridinger J. Rapid *In vivo* validation of HDAC inhibitor-based treatments in neuroblastoma zebrafish xenografts. Pharm (Basel) (2020) 13(11):345. doi: 10.3390/ph13110345 PMC7692187 33121173

[97] Timme N Han Y Liu S Yosief HO García HD Bei Y. Small-molecule dual PLK1 and BRD4 inhibitors are active against preclinical models of pediatric solid tumors. Transl Oncol (2020) 13(2):221–32. doi: 10.1016/j.tranon.2019.09.013 PMC6931204 31869746

[98] Ham J Costa C Sano R Lochmann TL Sennott EM Patel NU. Exploitation of the apoptosis-primed state of MYCN-amplified neuroblastoma to develop a potent and specific targeted therapy combination. Cancer Cell (2016) 29(2):159–72. doi: 10.1016/j.ccell.2016.01.002 PMC4749542 26859456

[99] Decaesteker B Denecker G Van Neste C Dolman EM Van Loocke W Gartlgruber M. TBX2 is a neuroblastoma core regulatory circuitry component enhancing MYCN/FOXM1 reactivation of DREAM targets. Nat Commun (2018) 9(1):4866. doi: 10.1038/s41467-018-06699-9 30451831 PMC6242972

[100] Sano R Krytska K Larmour CE Raman P Martinez D Ligon GF. An antibody-drug conjugate directed to the ALK receptor demonstrates efficacy in preclinical models of neuroblastoma. Sci Transl Med (2019) 11(483):eaau9732. doi: 10.1126/scitranslmed.aau9732 30867324 PMC10023134

[101] Quinn CH Beierle AM Hutchins SC Marayati R Bownes LV Stewart JE. Targeting high-risk neuroblastoma patient-derived xenografts with oncolytic virotherapy. Cancers (2022) 14(3):762. doi: 10.3390/cancers14030762 35159029 PMC8834037

[102] Barry WE Jackson JR Asuelime GE Wu HW Sun J Wan Z. Activated natural killer cells in combination with anti-GD2 antibody dinutuximab improve survival of mice after surgical resection of primary neuroblastoma. Clin Cancer Res an Off J Am Assoc Cancer Res (2019) 25(1):325–33. doi: 10.1158/1078-0432.CCR-18-1317 PMC6320320 30232225

[103] Nguyen R Moustaki A Norrie JL Brown S Akers WJ Shirinifard A. Interleukin-15 enhances anti-GD2 antibody-mediated cytotoxicity in an orthotopic PDX model of neuroblastoma. Clin Cancer Res an Off J Am Assoc Cancer Res (2019) 25(24):7554–64. doi: 10.1158/1078-0432.CCR-19-1045 PMC6911623 31455682

[104] Nguyen R Zhang X Sun M Abbas S Seibert C Kelly MC. Anti-GD2 antibodies conjugated to IL15 and IL21 mediate potent antitumor cytotoxicity against neuroblastoma. Clin Cancer Res an Off J Am Assoc Cancer Res (2022) 28(17):3785–96. doi: 10.1158/1078-0432.CCR-22-0717 PMC9444978 35802683

[105] Monterrubio C Paco S Olaciregui NG Pascual-Pasto G Vila-Ubach M Cuadrado-Vilanova M. Targeted drug distribution in tumor extracellular fluid of GD2-expressing neuroblastoma patient-derived xenografts using SN-38-loaded nanoparticles conjugated to the monoclonal antibody 3F8. J Control Release (2017) 255:108–19. doi: 10.1016/j.jconrel.2017.04.016 PMC5564453 28412222

[106] Langenberg KPS Looze EJ Molenaar JJ. The landscape of pediatric precision oncology: Program design, actionable alterations, and clinical trial development. Cancers (2021) 13(17):4324. doi: 10.3390/cancers13174324 34503139 PMC8431194

[107] Parsons DW Janeway KA Patton DR Winter CL Coffey B Williams PM. Actionable tumor alterations and treatment protocol enrollment of pediatric and young adult patients with refractory cancers in the national cancer institute-children's oncology group pediatric MATCH trial. J Clin Oncol Off J Am Soc Clin Oncol (2022) 40(20):2224–34. doi: 10.1200/JCO.21.02838 PMC9273376 35353553

[108] van Tilburg CM Pfaff E Pajtler KW Langenberg KPS Fiesel P Jones BC. The pediatric precision oncology INFORM registry: Clinical outcome and benefit for patients with very high-evidence targets. Cancer discovery (2021) 11(11):2764–79. doi: 10.1158/2159-8290.CD-21-0094 PMC9414287 34373263

[109] Worst BC van Tilburg CM Balasubramanian GP Fiesel P Witt R Freitag A. Next-generation personalised medicine for high-risk paediatric cancer patients - the INFORM pilot study. Eur J Cancer (2016) 65:91–101. doi: 10.1016/j.ejca.2016.06.009 27479119

[110] Newman S Nakitandwe J Kesserwan CA Azzato EM Wheeler DA Rusch M. Genomes for kids: The scope of pathogenic mutations in pediatric cancer revealed by comprehensive DNA and RNA sequencing. Cancer discovery (2021) 11(12):3008–27. doi: 10.1158/2159-8290.CD-20-1631 PMC8783930 34301788

[111] Houghton PJ Morton CL Tucker C Payne D Favours E Cole C. The pediatric preclinical testing program: Description of models and early testing results. Pediatr Blood cancer (2007) 49(7):928–40. doi: 10.1002/pbc.21078 17066459

[112] Zwaan CM Kearns P Caron H Verschuur A Riccardi R Boos J. The role of the 'innovative therapies for children with cancer' (ITCC) European consortium. Cancer Treat Rev (2010) 36(4):328–34. doi: 10.1016/j.ctrv.2010.02.008 20231057

[113] Lau LMS Mayoh C Xie J Barahona P MacKenzie KL Wong M. *In vitro* and in vivo drug screens of tumor cells identify novel therapies for high-risk child cancer. EMBO Mol Med (2022) 14(4):e14608. doi: 10.15252/emmm.202114608 34927798 PMC8988207

[114] Langenberg K Dolman E Molenaar J. Abstract A40: Integration of high-throughput drug screening on patient-derived organoids into pediatric precision medicine programs: The future is now! Cancer Res (2020) 80(s):A40. doi: 10.1158/1538-7445.PEDCA19-A40

[115] ElHarouni D Berker Y Peterziel H Gopisetty A Turunen L Kreth S. iTReX: Interactive exploration of mono- and combination therapy dose response profiling data. Pharmacol Res (2022) 175:105996. doi: 10.1016/j.phrs.2021.105996 34848323

[116] Berker Y ElHarouni D Peterziel H Fiesel P Witt O Oehme I. Patient-by-Patient deep transfer learning for drug-response profiling using confocal fluorescence microscopy of pediatric patient-derived tumor-cell spheroids. IEEE Trans Med Imaging (2022) 41(12):3981–99. doi: 10.1109/TMI.2022.3205554 36099221

[117] Tadeo I Berbegall AP Castel V García-Miguel P Callaghan R Påhlman S. Extracellular matrix composition defines an ultra-high-risk group of neuroblastoma within the high-risk patient cohort. Br J cancer (2016) 115(4):480–9. doi: 10.1038/bjc.2016.210 PMC4985353 27415013

[118] Burgos-Panadero R El Moukhtari SH Noguera I Rodríguez-Nogales C Martín-Vañó S Vicente-Munuera P. Unraveling the extracellular matrix-tumor cell interactions to aid better targeted therapies for neuroblastoma. Int J Pharm (2021) 608:121058. doi: 10.1016/j.ijpharm.2021.121058 34461172

[119] Martinov T McKenna KM Tan WH Collins EJ Kehret AR Linton JD. Building the next generation of humanized hemato-lymphoid system mice. Front Immunol (2021) 12:643852. doi: 10.3389/fimmu.2021.643852 33692812 PMC7938325

[120] Rongvaux A Willinger T Martinek J Strowig T Gearty SV Teichmann LL. Development and function of human innate immune cells in a humanized mouse model. Nat Biotechnol (2014) 32(4):364–72. doi: 10.1038/nbt.2858 PMC4017589 24633240

[121] Nguyen R Patel AG Griffiths LM Dapper J Stewart EA Houston J. Next-generation humanized patient-derived xenograft mouse model for pre-clinical antibody studies in neuroblastoma. Cancer Immunol Immunother (2021) 70(3):721–32. doi: 10.1007/s00262-020-02713-6 PMC8276267 32915319

[122] Drost J Clevers H. Organoids in cancer research. Nat Rev Cancer (2018) 18(7):407–18. doi: 10.1038/s41568-018-0007-6 29692415

[123] Tuveson D Clevers H. Cancer modeling meets human organoid technology. Sci (New York NY) (2019) 364(6444):952–5. doi: 10.1126/science.aaw6985 31171691

[124] Kholosy WM Derieppe M van den Ham F Ober K Su Y Custers L. Neuroblastoma and DIPG organoid coculture system for personalized assessment of novel anticancer immunotherapies. J Pers Med (2021) 11(9):869. doi: 10.3390/jpm11090869 34575646 PMC8466534

[125] Jenkins RW Aref AR Lizotte PH Ivanova E Stinson S Zhou CW. Ex vivo profiling of PD-1 blockade using organotypic tumor spheroids. Cancer discovery (2018) 8(2):196–215. doi: 10.1158/2159-8290.CD-17-0833 29101162 PMC5809290

[126] Cui X Ma C Vasudevaraja V Serrano J Tong J Peng Y. Dissecting the immunosuppressive tumor microenvironments in glioblastoma-on-a-Chip for optimized PD-1 immunotherapy. Elife (2020) 9:e52253. doi: 10.7554/eLife.52253 32909947 PMC7556869

[127] Nothdurfter D Ploner C Coraça-Huber DC Wilflingseder D Müller T Hermann M. 3D bioprinted, vascularized neuroblastoma tumor environment in fluidic chip devices for precision medicine drug testing. Biofabrication (2022) 14(3):035002. doi: 10.1088/1758-5090/ac5fb7 35333193

[128] Yu JL Chan S Fung MK Chan GC. Mesenchymal stem cells accelerated growth and metastasis of neuroblastoma and preferentially homed towards both primary and metastatic loci in orthotopic neuroblastoma model. BMC cancer (2021) 21(1):393. doi: 10.1186/s12885-021-08090-2 33838662 PMC8035760

[129] Grigoryan A Zacharaki D Balhuizen A Côme CR Garcia AG Hidalgo Gil D. Engineering human mini-bones for the standardized modeling of healthy hematopoiesis, leukemia, and solid tumor metastasis. Sci Trans Med (2022) 14(666):eabm6391. doi: 10.1126/scitranslmed.abm6391 36223446

